# Supplementary material for: Application of 2D NMR Spectroscopy in Combination with Chemometric Tools for Classification of Natural Lignins
Source: Int J Mol Sci. 2023 Aug 3;24(15):12403. doi: 10.3390/ijms241512403 (PMC10419134; doi:10.3390/ijms241512403)
Supplement: Supplementary file 1 [file ijms-24-12403-s001.zip › ijms-2509674-supplementary.pdf]

Supplementary File

Application of 2D NMR Spectroscopy in Combination with Chemometric Tools for Classification of Natural Lignins

Anna V. Faleva *, Ilya A. Grishanovich, Nikolay V. Ul'yanovskii and Dmitry S. Kosyakov *

Laboratory of Natural Compounds Chemistry and Bioanalytics, Core Facility Center "Arktika", M.V. Lo-monosov Northern (Arctic) Federal University, Northern Dvina Emb. 17, 163002 Arkhangelsk, Russia; grilandr@gmail.com (I.A.G.); n.ulyanovsky@narfu.ru (N.V.U.)

* Correspondence: a.bezumova@narfu.ru (A.V.F.); d.kosyakov@narfu.ru (D.S.K.)

Contents:

Figure S1. Residual dispersion curves: PCA of all lignins (left); analysis of coniferous lignins (right)

Figure S2. A part of PC1 load plot in the form of a 2D NMR spectrum.

Figure S3. A part of PC2 load plot in the form of a 2D NMR spectrum.

Figure S4. A part of PC3 load plot in the form of a 2D NMR spectrum.

Figure S5. A part of PC4 load plot in the form of a 2D NMR spectrum.

Figure S6. A part of PC1 load plot of softwood lignin in the form of a 2D NMR spectrum.

Figure S7. A part of PC2 load plot of softwood lignin in the form of a 2D NMR spectrum.

Figure S8. A part of PC7 load plot of softwood lignin in the form of a 2D NMR spectrum.

Table S1. Data on the effect of substructures based on load spectra in the analysis of lignins isolated from coniferous wood species

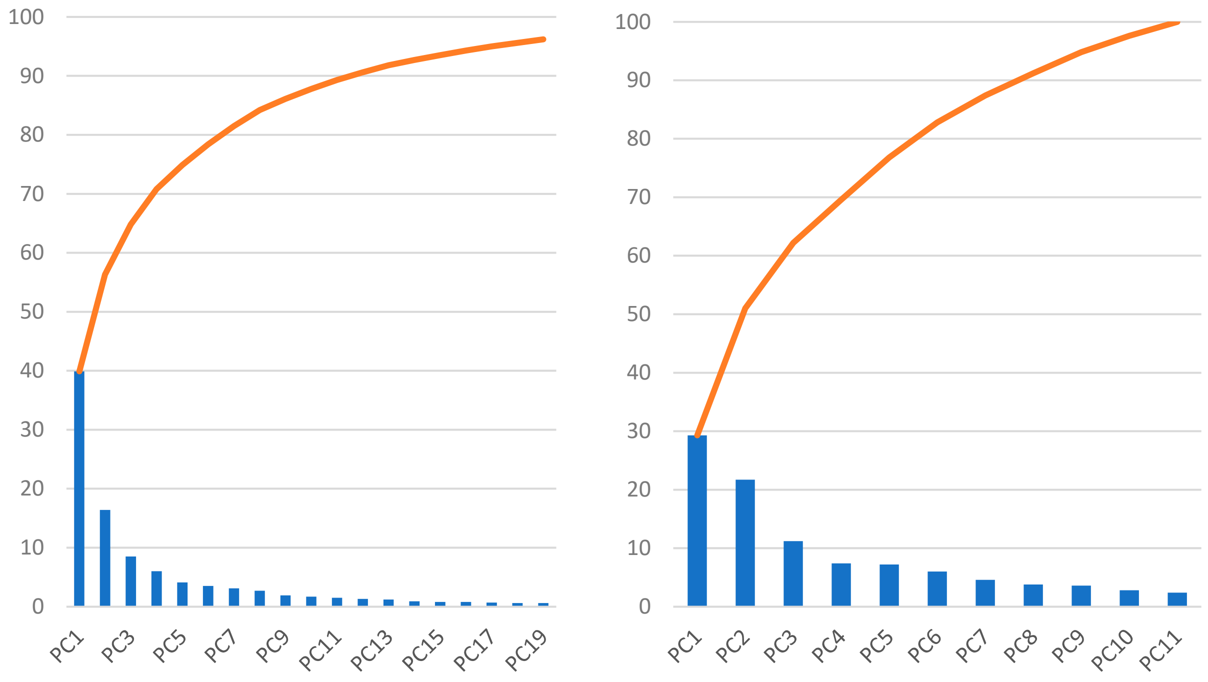

Figure S1. Residual dispersion curves: PCA of all lignins (left); analysis of coniferous lignins (right)

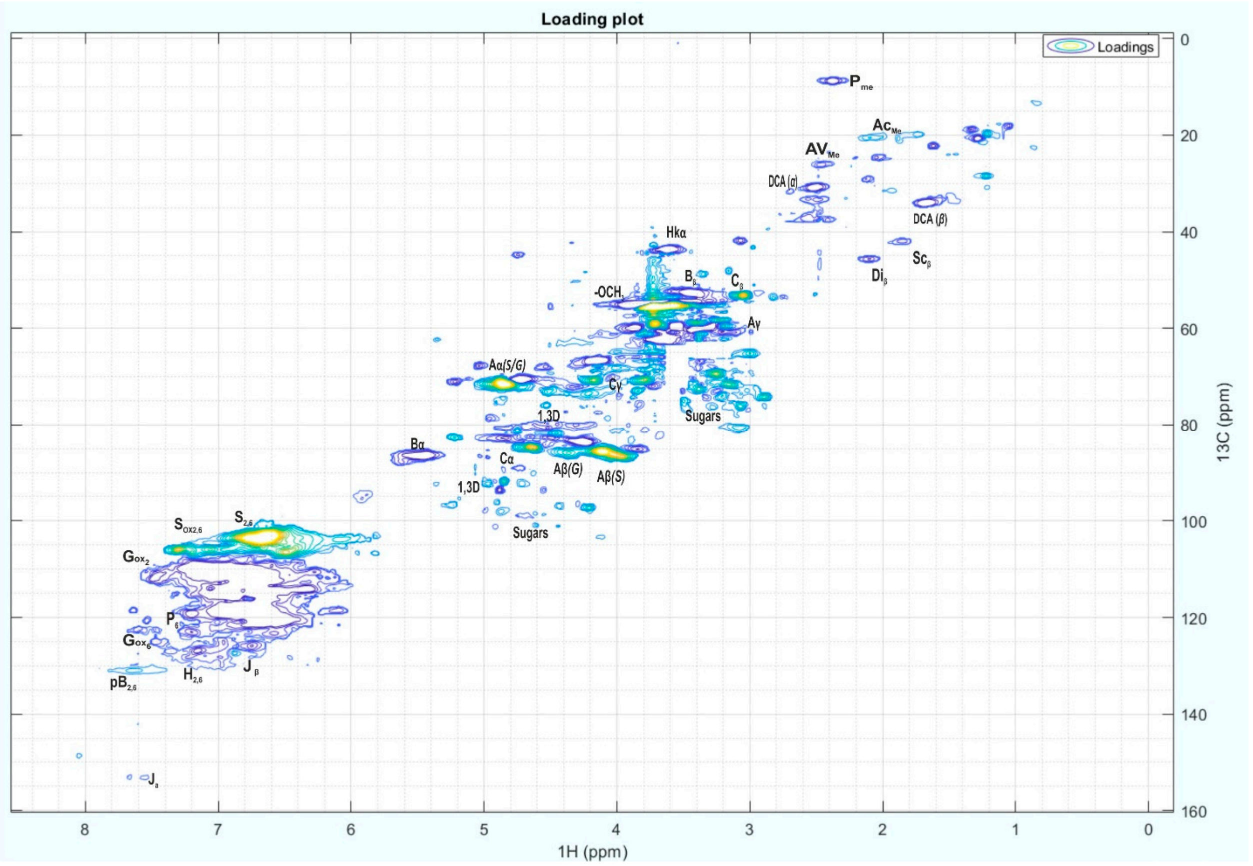

Positive cross-peaks (yellow) correlate with samples with positive values along PC1 and vice versa negative cross-peaks (blue) correlate with samples with a negative value along the PC1 axis

Figure S2. A part of PC1 load plot in the form of a 2D NMR spectrum.

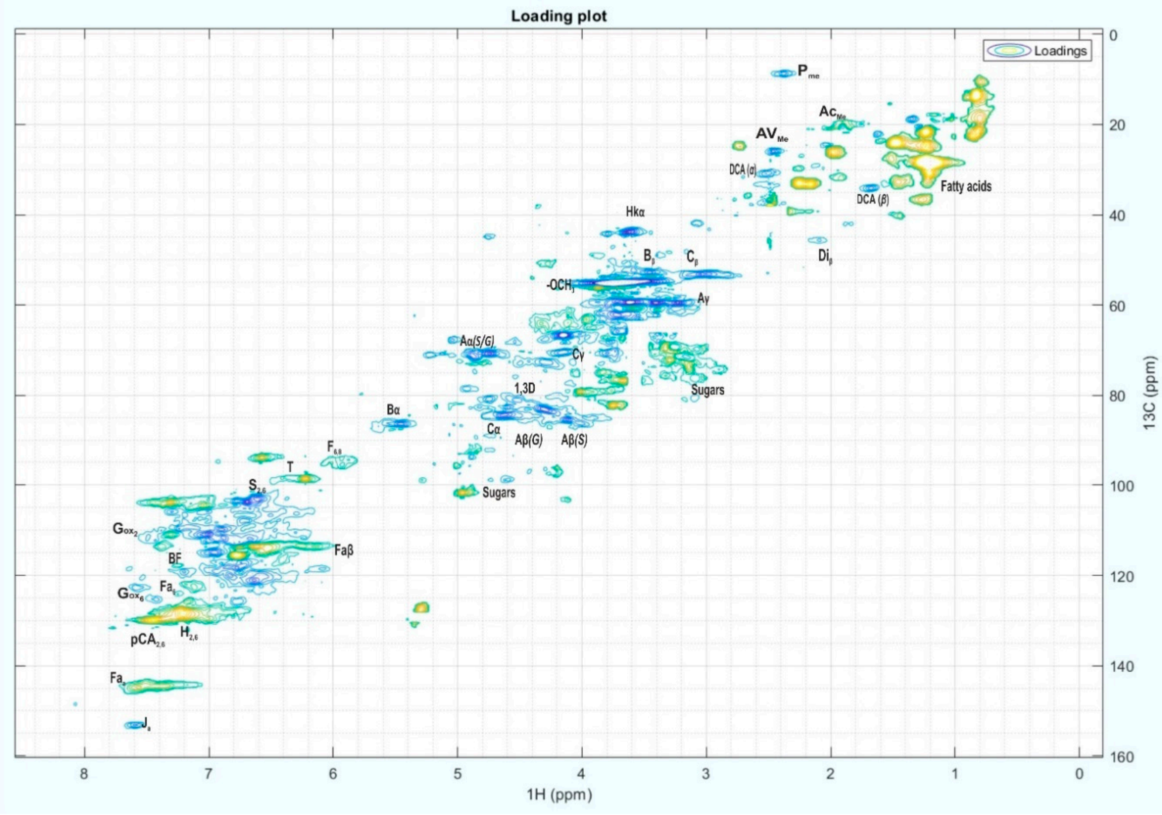

Positive cross-peaks (yellow) correlate with samples with positive values along PC2 and vice versa negative cross-peaks (blue) correlate with samples with a negative value along the PC2 axis

Figure S3. A part of PC2 load plot in the form of a 2D NMR spectrum.

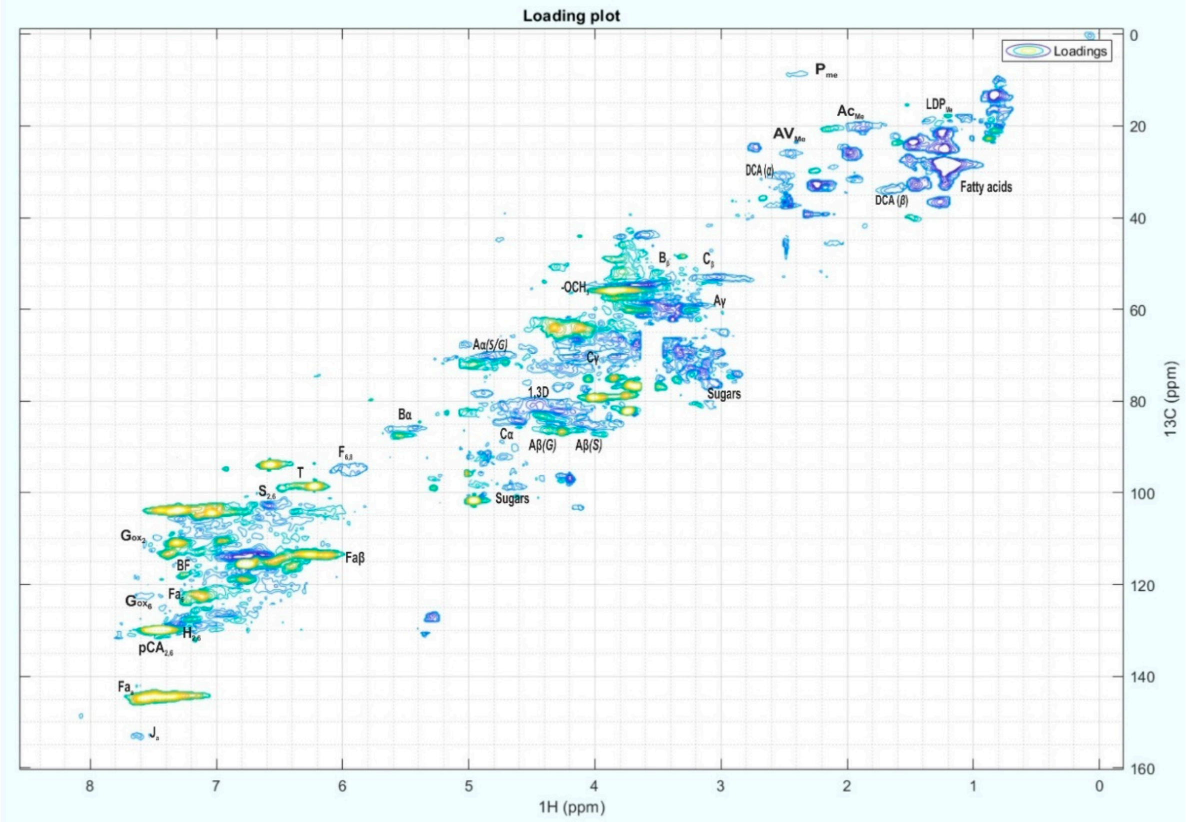

Positive cross-peaks (yellow) correlate with samples with positive values along PC3 and vice versa negative cross-peaks (blue) correlate with samples with a negative value along the PC3 axis

Figure S4. A part of PC3 load plot in the form of a 2D NMR spectrum.

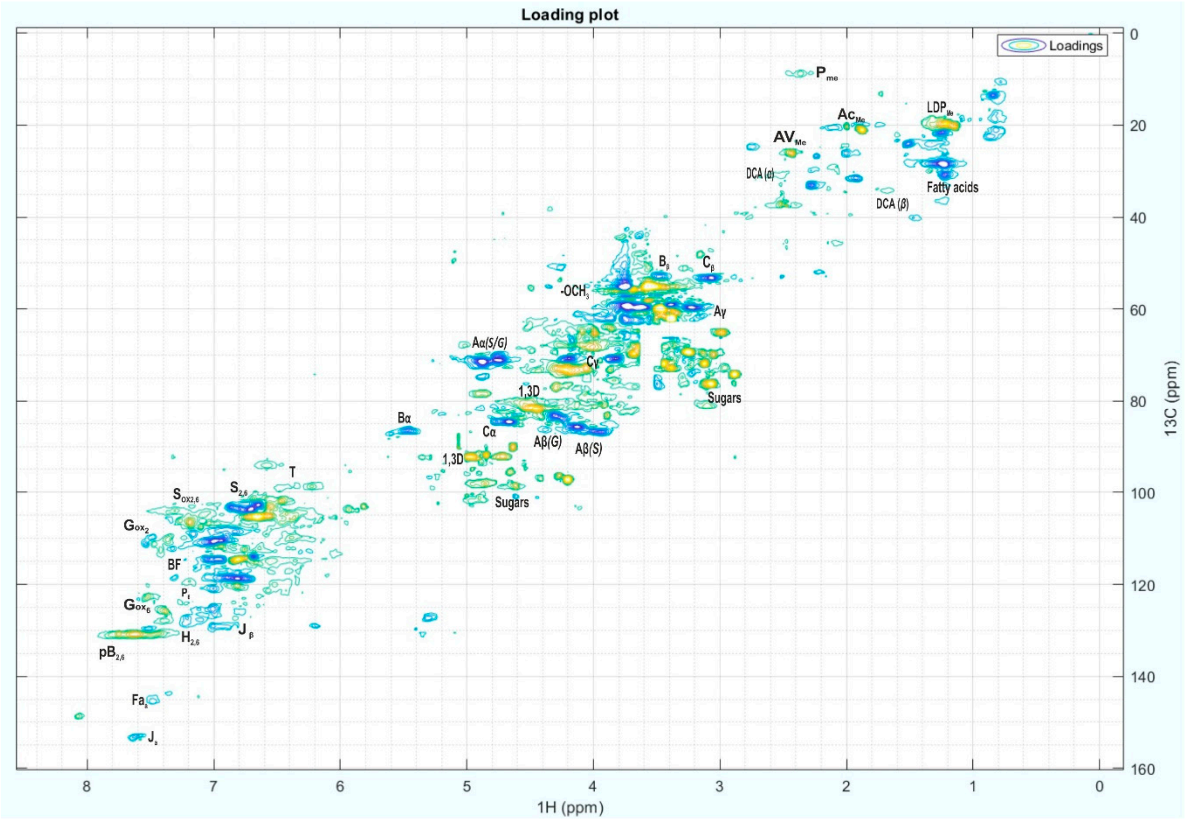

Positive cross-peaks (yellow) correlate with samples with positive values along PC4 and vice versa negative cross-peaks (blue) correlate with samples with a negative value along the PC4 axis

Figure S5. A part of PC4 load plot in the form of a 2D NMR spectrum.

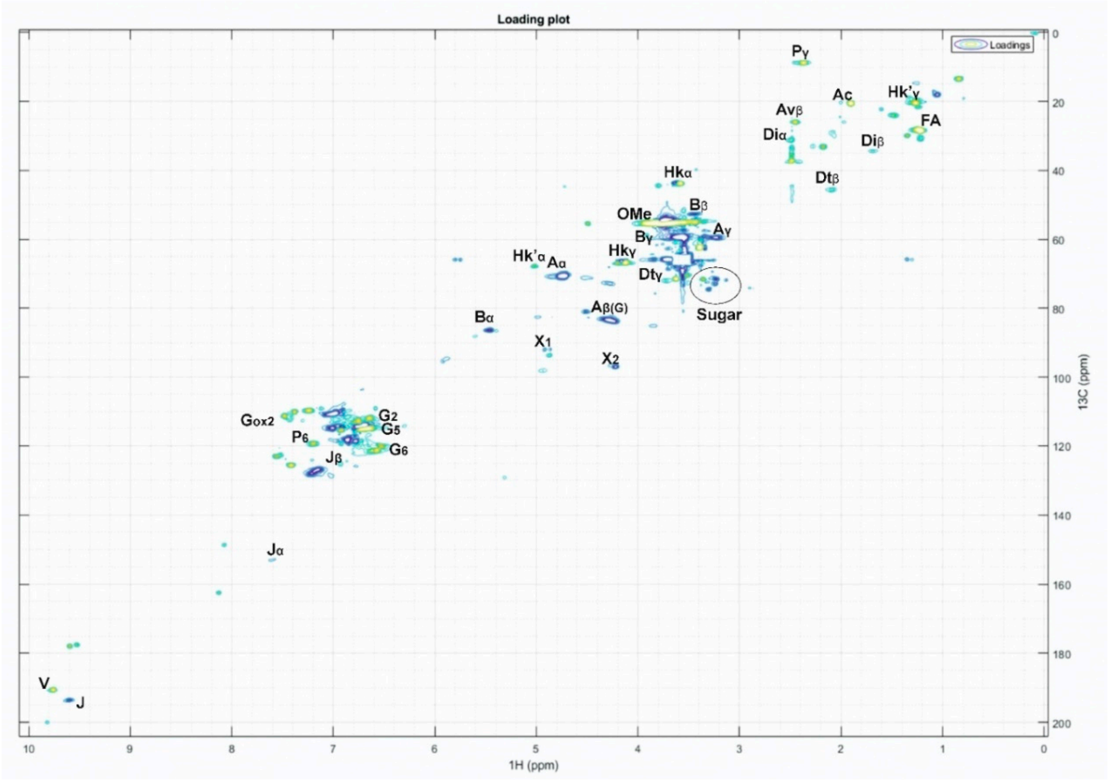

Positive cross-peaks (yellow) correlate with samples with positive values along PC1 and vice versa negative cross-peaks (blue) correlate with samples with a negative value along the PC1 axis

Figure S6. A part of PC1 load plot of softwood lignin in the form of a 2D NMR spectrum.

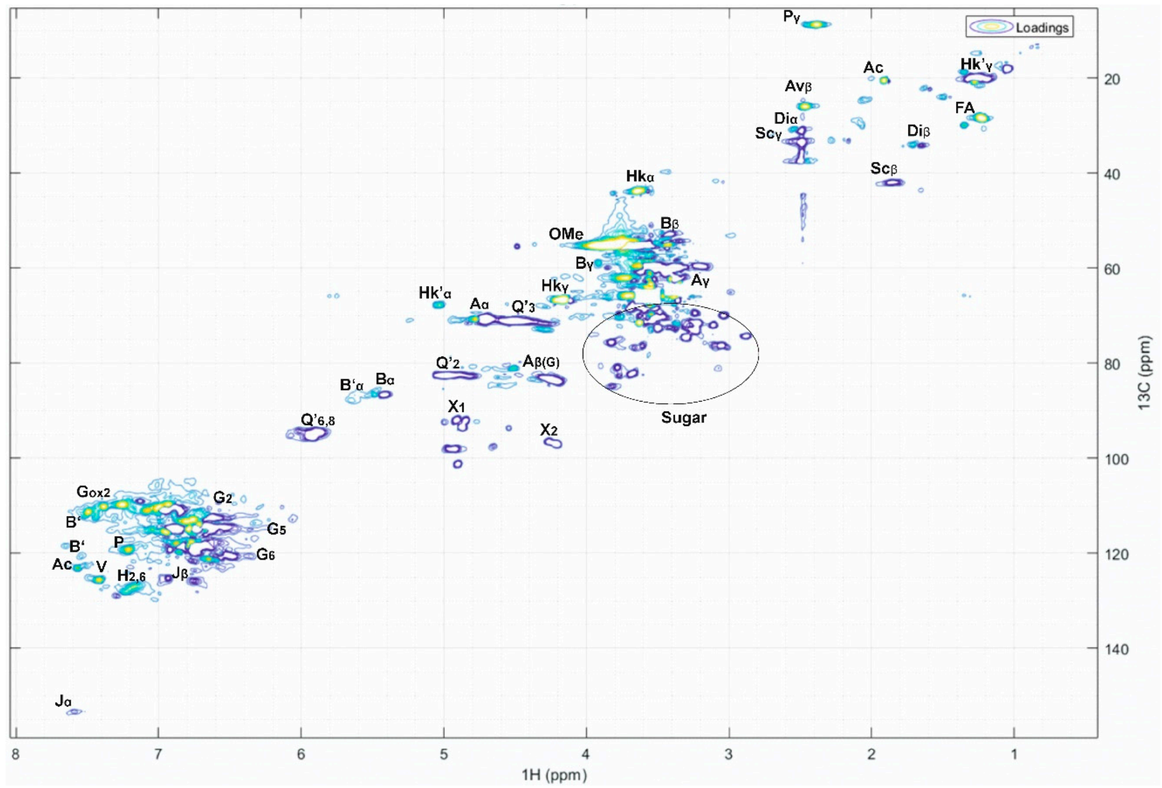

Positive cross-peaks (yellow) correlate with samples with positive values along PC2 and vice versa negative cross-peaks (blue) correlate with samples with a negative value along the PC2 axis

Figure S7. A part of PC2 load plot of softwood lignin in the form of a 2D NMR spectrum.

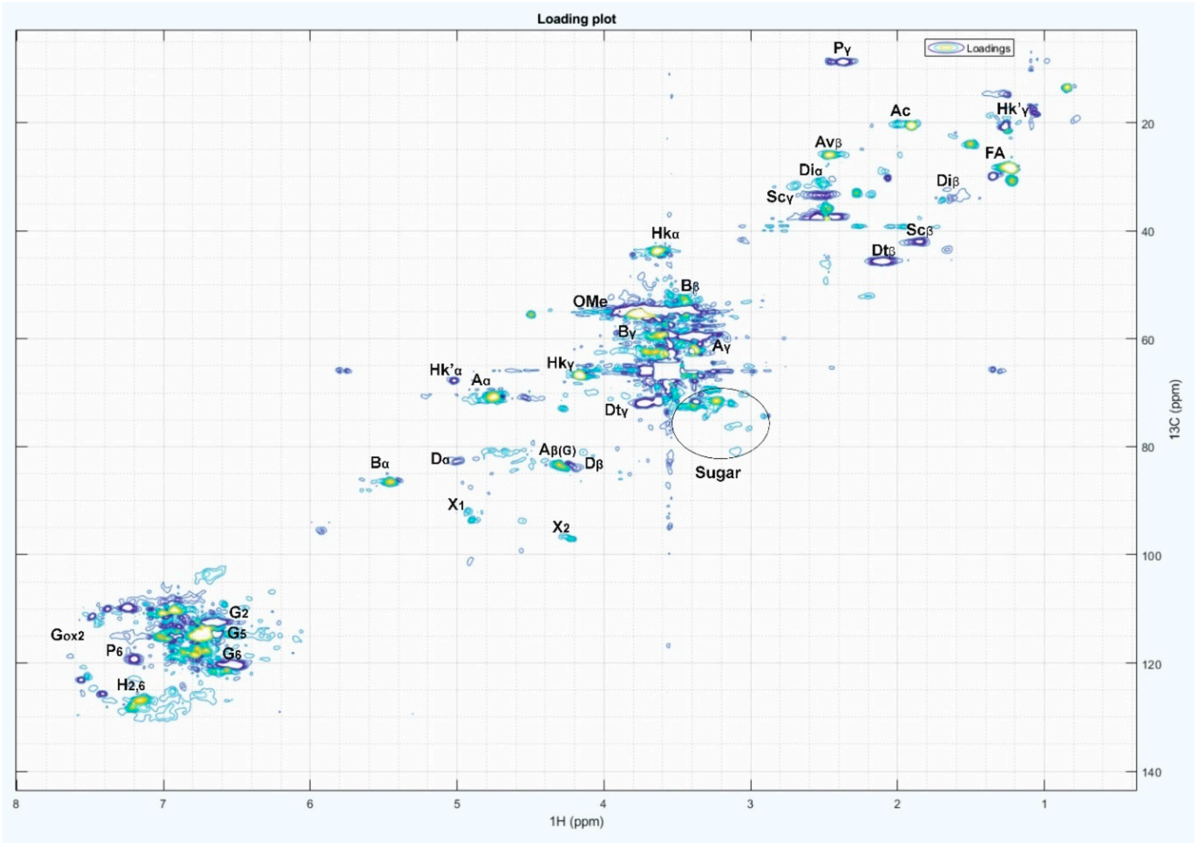

Positive cross-peaks (yellow) correlate with samples with positive values along PC7 and vice versa negative cross-peaks (blue) correlate with samples with a negative value along the PC7 axis

Figure S8. A part of PC7 load plot of softwood lignin in the form of a 2D NMR spectrum.

Table S1. Data on the effect of substructures based on load spectra in the analysis of lignins isolated from coniferous wood species

Name of substructures	Label	PC1	PC2	PC7
		29.3, %	21.7, %	4.6, %
Main substructures				
p-Hydroxyphenyl PPU	H	-	+	+
Syringyl PPU	S	n/d	n/d	+
β-aryl ether (G)	A	-	-	+
Phenylcoumarone	B	-	+-	+
Secoisolariciresinol	Sc	n/d	-	-
Dibenzodioxocin	D	-	-	n/d
Pinoresinol	C	n/d	+	n/d
Dihydroconiferyl alcohol	DCA	+	+/-	n/d
Coniferyl aldehyde	J	-	-	+
Substructures formed during extraction				
methyl substituted phenylcoumarone	P	+	+	-
3,4-divanylyltetrahydrofuran	*Di*	+	n/d	-
Hibbert's ketone	Hk	+	+	+
α- hydroxypropiovanilone	Hk'	+	+	-
Acetovanillone	AV	+	+	+
Vanillin	V	+	+	-
Other				
Sugars	Sugars	-	-	+
Fatty acids (+ Acetate)	Fatty acids (+Ac)	+	+	+
Taxifolin	F	-	-	-
